# Influence of Myrmecophytic *Acacia drepanolobium* on the Composition and Growth of Surrounding Herbaceous Vegetation

**DOI:** 10.1002/ece3.71500

**Published:** 2025-05-25

**Authors:** Julius C. Karugu, Duncan M. Kimuyu, David Kenfack, Moshe Inbar

**Affiliations:** ^1^ Department of Natural Resources Karatina University Nyeri Kenya; ^2^ Mpala Research Center Laikipia Kenya; ^3^ Department of Evolutionary & Environmental Biology University of Haifa Haifa Israel; ^4^ Forest Global Earth Observatory‐Smithsonian Tropical Research Institute Washington DC USA

**Keywords:** community composition, facilitation, herbivory, Laikipia County, Mpala Research Center, nurse plants, whistling thorn acacia

## Abstract

Whistling thorn acacia (*Acacia* (*Vachellia*) *drepanolobium*) forms nearly monospecific stands among woody species in black cotton soils in East Africa arid highlands. The tree defends itself against large mammal herbivores with spinescence and symbiotic ants. While these defenses have been extensively studied, little is known about the extent to which *A. drepanolobium* defense may benefit other plants growing in close association. We examined variation in herbaceous vegetation height, biomass, and composition between areas underneath *A. drepanolobium* canopies and the adjacent matrix in both fenced herbivore exclosures and unfenced areas. In unfenced areas, there was more tall herbaceous vegetation and biomass underneath tree canopies than away from tree canopies, while these differences were not significant in fenced exclosures. Both height and biomass of understory vegetation were negatively correlated with *A. drepanolobium* canopy height. Species richness was higher underneath tree canopies in both fenced and unfenced locations. In the unfenced locations, species evenness was lower underneath tree canopies than in the surrounding matrix, but the opposite was true in the fenced herbivore exclosures. The differences in herbaceous vegetation composition (Bray–Curtis dissimilarity index) between underneath tree and off tree locations were more pronounced in the unfenced areas than within the fenced herbivore exclosures. Our findings suggest that highly defended trees may moderate herbivore effects on herbaceous vegetation. To the extent that herbaceous vegetation underneath trees experiences protection from herbivory, such refugia microhabitats may serve as recolonization nuclei in attempts to restore chronically overgrazed systems.

## Graphical abstract

In areas facing intense herbivory pressure, heavily defended trees like Acacia drepanolobium may serve as nurse plants, creating refugia microhabitats for the growth of vulnerable plants. Such refugia habitats promote diversity across the landscape and may also serve as recolonization nuclei for rare species.

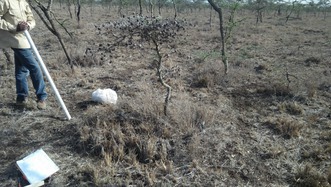

## 1 Introduction

Spatial associations between plants strongly influence community structure and composition. While competition has been the most studied spatial association process, facilitative interactions among plants are common, especially in resource‐limited environments (Padilla and Pugnaire 2006). Among the well‐known benefits of spatial associations include ‘nursing syndrome’ where stress‐resistant species facilitate growth of heterospecific neighbours underneath their canopies (Bruno et al. 2003; Callaway et al. 2002). Nurse plants possess specific traits that confer them the ability to survive limiting environmental or biotic conditions. Once established, they provide benefits to other plants, such as ameliorating extreme conditions, improving resource availability, or protecting against herbivory. Beyond these benefits, nurse plants may drive a cascade of benefits on ecosystem functions, including influencing moisture regimes (Ruwanza 2019), promoting accumulation of organic carbon and nutrients in soils (Mitchley et al. 1996; Padilla and Pugnaire 2006; Ren et al. 2008; Ruwanza 2019), stabilising soils (Valiente‐Banuet and Ezcurra 1991), and increasing resilience of biotic communities (Aguiar and Sala 1994).

In East Africa's arid highlands, *Acacia* (*Vachellia*) *drepanolobium* forms nearly monospecific stands among woody species on black cotton soil (Goheen et al. 2004; Kenfack et al. 2021; Young et al. 1996). Black cotton soil has a high clay content and undergoes shrink‐swell cycles, forming deep cracks during the dry season (DeCarlo and Caylor 2019) and becoming waterlogged during the rainy season. These stressful conditions, coupled with herbivory, filter out most of the other plant species (Pringle et al. 2016; Young et al. 1997). *Acacia drepanolobium* survives herbivory through investment in defense by four symbiotic ant species, in addition to spinescence. The four ant species, *Crematogaster mimosae* , *C. nigriceps* , *C. sjostedti* , and *Tetraponera penzigi* , form exclusive colonies within an individual tree or multiple adjacent trees (Madden and Young 1992). The ants defend the host trees by swarming and biting herbivores (Goheen and Palmer 2010; Palmer et al. 2000). While this acacia‐ant mutualism has been extensively studied, little is known about the extent to which *A. drepanolobium* defense may benefit other plants growing in close association.

We compared herbaceous vegetation height (index of grazing pressure), biomass, and species composition underneath *A. drepanolobium* canopies with vegetation in the surrounding matrix. To tease apart the effect of herbivory by large mammals, we conducted similar comparisons in fenced herbivore exclosures plots. Additionally, we tested the influence of canopy height (height of the lowest branch) on the ability to nurse plants underneath their canopies. We hypothesized that, if *A. drepanolobium* protects understory herbaceous vegetation from herbivory, (i) underneath the trees, herbaceous vegetation would be taller and have higher biomass than in the adjacent matrix, (ii) trees with canopies close to the ground would protect more biomass compared to trees with higher canopies, (iii) the understory herbaceous vegetation community underneath the tree canopies would be more diverse than the surrounding matrix, (iv) these effects would be largely restricted to plots from which herbivores have not been excluded.

## 2 Materials and Methods

### 2.1 Study Area

**FIGURE 1:**
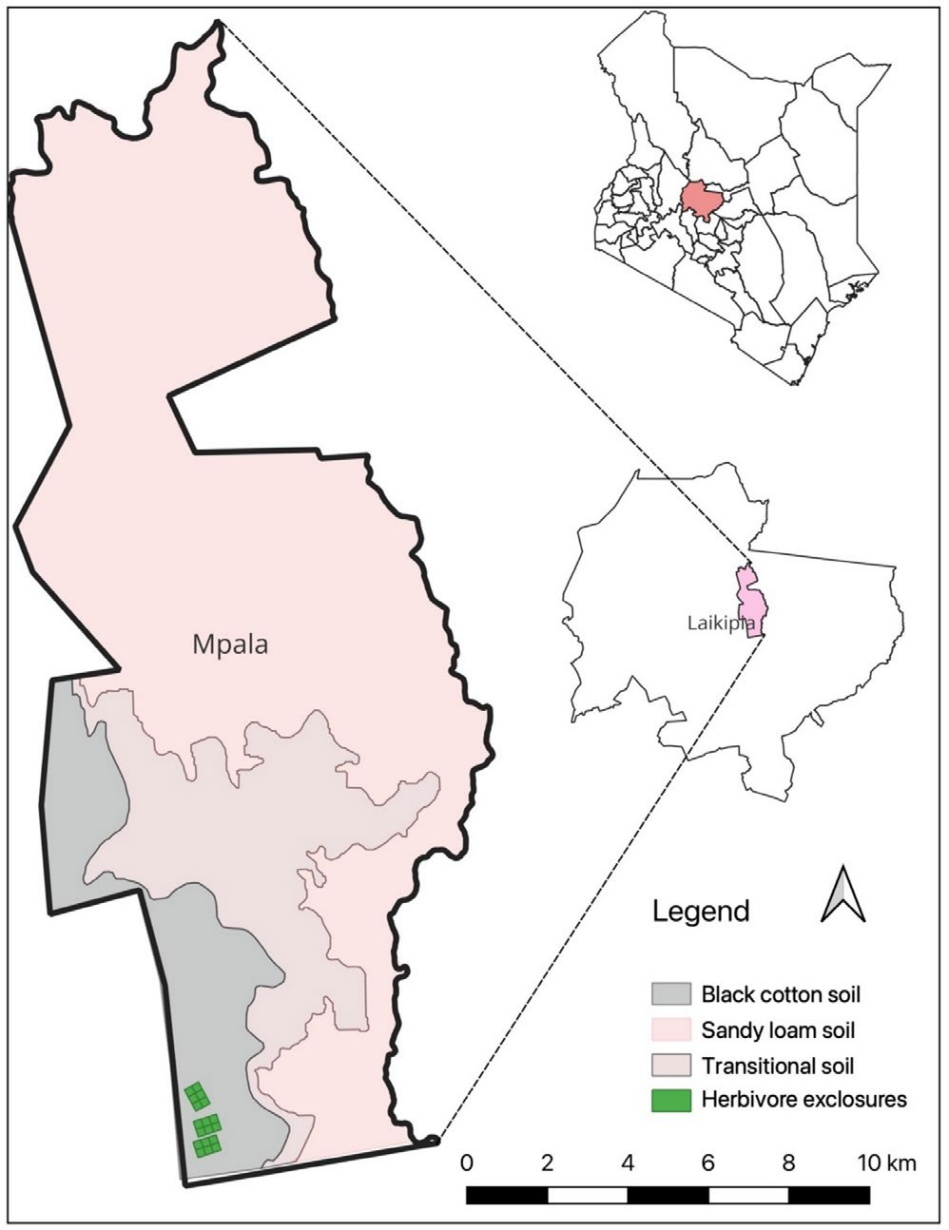
Map of the study area showing the location of the black cotton soil at Mpala.

The study was conducted at Mpala Research Centre (0.28 N, 36.86 E, 1800 M asl) in Laikipia County, Kenya, located on the dry leeward side of Mt. Kenya (Figure 1). Annual rainfall in the area averages 550–600 mm and is weakly trimodal, usually with a distinct dry season from December to March (Kimuyu et al. 2014). Study plots were selected within homogeneous high clay black cotton soils (vertisols). Black cotton soils occur extensively throughout eastern and southern Africa. Whistling thorn acacia (*A. drepanolobium*) accounts for more than 97% of woody cover at the study site (Goheen et al. 2004; Kenfack et al. 2021; Young et al. 1996). Other woody species in the study site include *Balanites aegyptiaca* , *Rhus natalensis*, and *Cabada farinosa*. The understory herbaceous layer is dominated by the perennial grasses *Pennisetum stramineum*, *P. mezianum*, *Brachiaria lachnantha*, *Themeda triandra* , *and Lintonia nutans*. Common forbs include *Aspilia pleuriseta*, *Commelina spp*., *Solanum* spp., *Pseudognaphalium* sp., *Aerva lanata* , *Dyscoriste radicans*, and *Rhynchosia holstii* (Kimuyu et al. 2017). The Mpala is managed for both wildlife conservation and livestock production. Cattle are the main grazers, which have been moderately stocked (0.10–0.15 cattle/ha) for the past several decades. Wild ungulates include Burchell's zebra ( *Equus burchelli* Grey), Jackson's hartebeest (*Alcelaphus buselaphus jacksoni* Pallas), steenbuck ( *Raphicerus campestris* Thunberg), Grant's gazelle (*Gazella grand* Brooke), Beisa oryx ( *Oryx beisa* Ruppell), eland ( *Taurotragus oryx* Pallas), elephant ( *Loxodonta africana* Blumenbach), and giraffe ( *Giraffa camelopardalis* L.) (Kimuyu et al. 2017).

### 2.2 Study Species

*Acacia drepanolobium* typically grows between 3 and 6 m in height, but can reach up to 10 m under favorable conditions. It commonly develops a rounded, umbrella‐like, or flattened crown, with a canopy diameter ranging from 2 to 5 m. The species generally exhibits a single, upright trunk, though multiple stems may arise from a single base if the tree has been damaged by browsing or fire. Primary branches emerge at relatively low angles from the trunk, extending outward before slightly drooping at the tips, contributing to the characteristic flattened crown. Secondary branches are shorter and arise in an irregular pattern, often resulting in a dense, bushy appearance in younger trees. The tree's bipinnate leaves consist of numerous small, oblong leaflets arranged alternately along the branches. These leaves are typically concentrated toward the outer canopy, creating a semi‐open structure that permits some light penetration to the understory. As a result, the dappled sunlight beneath *A. drepanolobium* may influence the growth of grasses and shrubs in its surroundings.

### 2.3 Sampling Design

To evaluate the extent to which *A. drepanolobium* may affect herbaceous vegetation, we sampled vegetation biomass and composition underneath tree canopies and in the adjacent open spaces. For tree canopy sites, we sampled areas under short (< 1 m tall) trees as well as areas under tall (> 2 m) trees. Further, we selected control sites within an adjacent herbivore exclosure, Kenya Long‐term Exclosure Experiment (KLEE, Young et al. 1997) where all large wild and domestic herbivores (> 15 kg) have been excluded over the last 27 years. Our sampling design included 150 locations outside the herbivore exclosures (50 underneath tall trees, 50 underneath short trees, and 50 away from tree canopies) and 60 control sites inside the herbivore exclosures (20 underneath tall trees, 20 underneath short trees, and 20 away from tree canopies). We avoided sampling in areas with other known sources of heterogeneity, termite mounds (Fox‐Dobbs et al. 2010; Sileshi et al. 2010) and previously occupied cattle bomas (Veblen 2012).

### 2.4 Herbaceous Vegetation Sampling

We used the canopy intercept method (Dunkerley 2000; Frank and McNaughton 1990) to assess vegetation composition. This method involves the number of contacts (“hits”) a lowered pin makes with each plant species. A 1‐m‐wide pin frame with 10 equally spaced pins was placed at three random locations within each sampling site and all pin hits by species were recorded. After conducting the herbaceous vegetation composition survey, the aboveground plant material within 1 × 1 m quadrats in the sampled sites was clipped. The clipped biomass was collected in bags, air‐dried to a constant weight, and weighed. For each of the sampled tree locations, we measured the height of the lowest point of the tree canopy, hereafter referred to as canopy height.

### 2.5 Data Analyses

From the pin frame data, we calculated species richness and evenness. We tested for significant differences in herbaceous vegetation height, biomass, species richness, and evenness between sampling sites using general linear mixed‐effect (GLMM) models in the lme4 package (Douglas Bates et al. 2015) in R program version 4.2.3 (Team 2020). We specified location (under tree and off tree), herbivore treatment (fenced and unfenced) and their interaction as the main effect, and each sampling site as a random intercept to account for potential spatial autocorrelation between adjacent sites. Further, we used general linear models to test for the relationship between both herbaceous vegetation height and biomass and tree canopy height, between fenced and unfenced areas. For all significant effects, we performed multiple comparisons using the *emmeans* package (Lenth et al. 2020).

## 3 Results

### 3.1 Variation in Herbaceous Vegetation Height and Biomass

**FIGURE 2:**
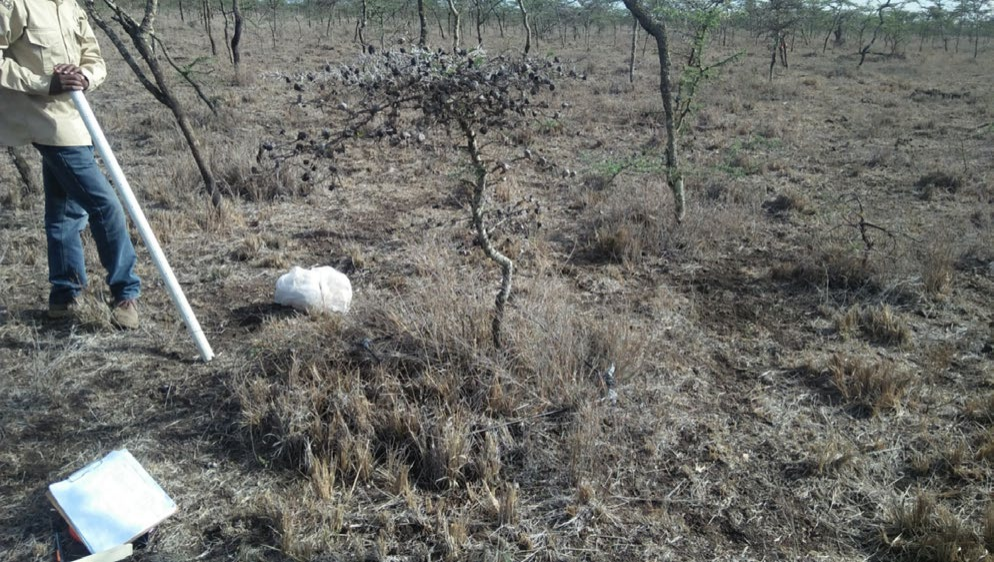
Tall grass underneath *acacia drepanolobium* tree. Areas farther from the tree canopy tend to have shorter grass and less biomass due to intensive grazing.

**FIGURE 3:**
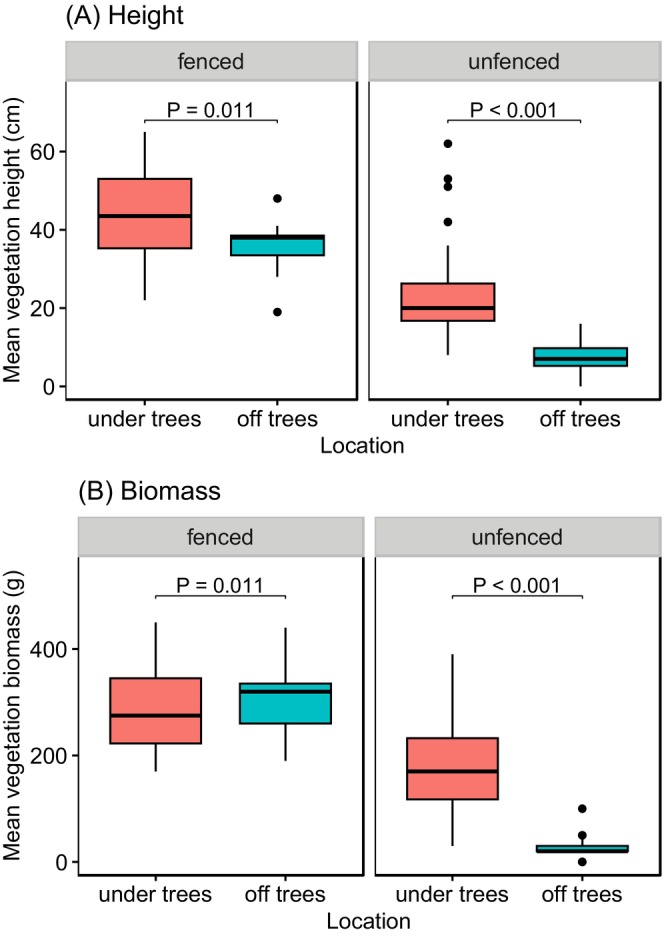
Variation in (A) height and (B) biomass of understory vegetation between under tree locations and off tree locations in both fenced and unfenced areas. The error bars depict standard error.

Herbaceous vegetation height and biomass were generally higher within the herbivore exclosures than in the unfenced areas, and underneath trees than away from tree canopies (Figure 2). The difference in herbaceous vegetation height between under tree and off‐tree locations was more prominent in unfenced areas than in the herbivore exclosures (tree × herbivore interaction: *χ* ^2^ = 4.80, *p* = 0.029). In the unfenced areas, herbaceous vegetation was 2.2 times taller underneath trees (22.3 ± 1.20 cm) than off trees (7.0 ± 1.70 cm), while within the herbivore exclosures, herbaceous vegetation underneath tree canopies was only 20% taller (43.4 ± 1.70 cm) than off tree canopies (35.9 ± 2.36 cm) (Figure 3a). Similarly, the difference in herbaceous vegetation biomass underneath trees and away from tree canopies depended on herbivore presence (tree × herbivore interaction: *χ* ^2^ = 38.03, *p* < 0.001). In the unfenced areas, herbaceous vegetation biomass was 5.9 times higher underneath tree canopies (178.9 ± 9.86 g) than away from the tree canopies (26.1 ± 14.00 g), while these differences were not significant within the herbivore exclosures (Figure 3b).

**FIGURE 4:**
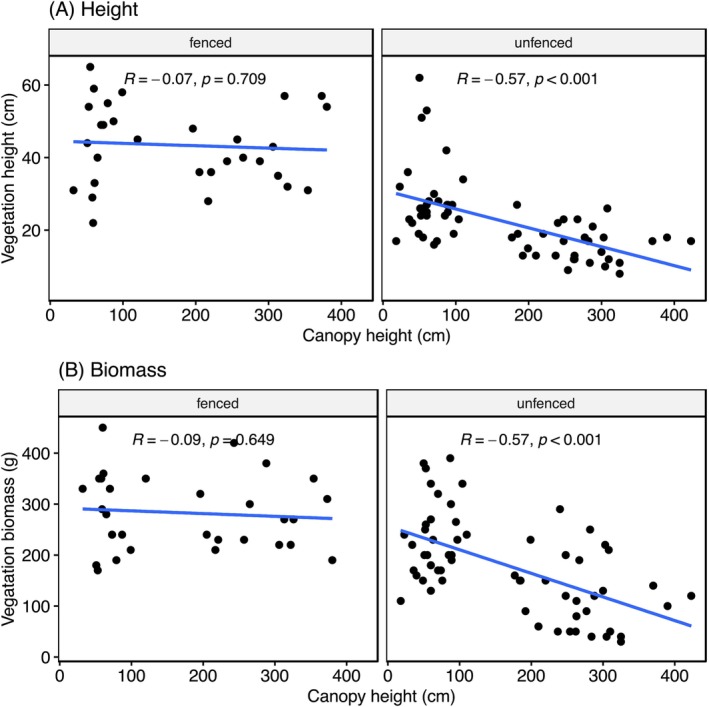
Influence of canopy height on understory vegetation (A) height and (B) biomass on both fenced and unfenced areas.

Across all sampling sites located under trees, effects of tree canopy height on both herbaceous vegetation height (*χ* ^2^ = 6.07, *p* = 0.014) and herbaceous biomass (*χ* ^2^ = 7.76, *p* = 0.005) depended on whether herbivores were fenced out or not. In unfenced plots, both vegetation height and biomass were highest under trees with canopies close to the ground and decreased with increasing canopy height (Figure 4). Within the herbivore exclosures, canopy height did not have a significant influence on either vegetation height or biomass (Figure 4).

### 3.2 Variation in Species Diversity and Composition

**FIGURE 5:**
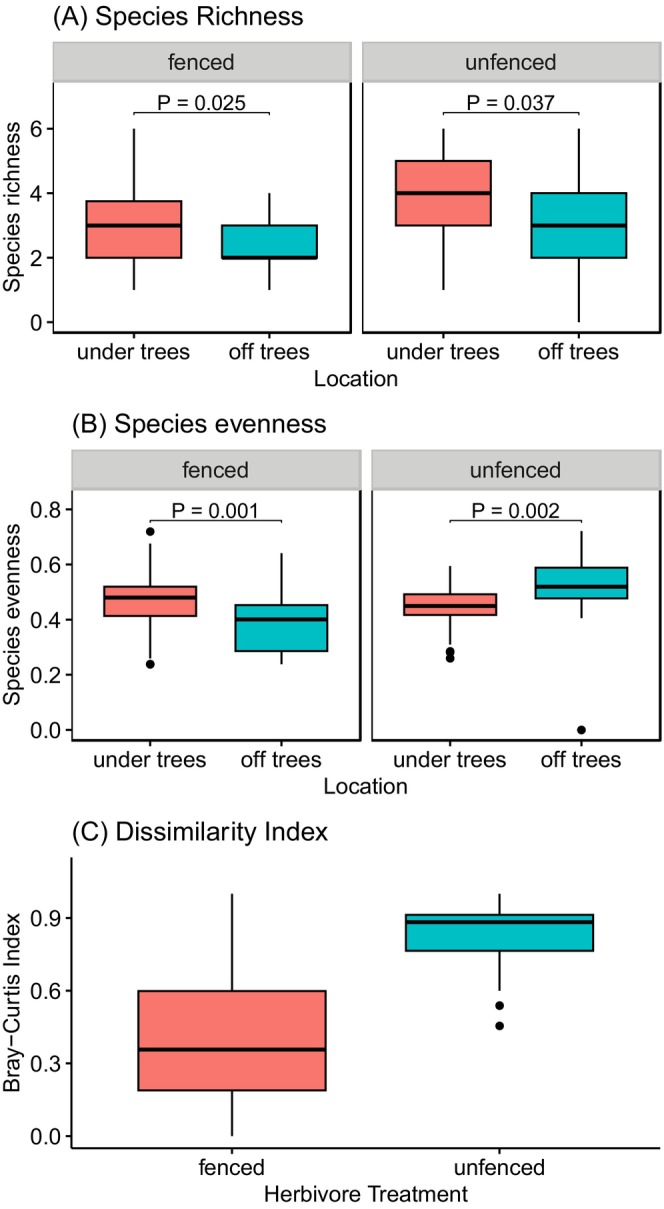
Variation in (A) herbaceous species diversity and (B) composition (Bray–Curtis dissimilarity) between under tree locations and off tree locations in both fenced and unfenced areas.

Herbaceous vegetation diversity and composition varied markedly across the sampling sites, depending on whether herbivores were allowed or not. In both fenced and unfenced areas, species richness was significantly higher underneath trees than away from tree canopies (*F* = 24.21, *p* < 0.001, Figure 5a). Species richness also varied across herbivore treatments, with fenced areas having higher richness than unfenced areas (*F* = 9.54, *p* = 0.002). There was a significant interaction effect of location and herbivore treatment on species evenness (*F* = 20.83, *p* < 0.001). Within the herbivore exclosures, under tree locations had 16% higher species evenness than off tree locations (Figure 5b). In the unfenced areas, species evenness was 14% higher in the off‐tree locations than underneath trees. We found greater differences in species assemblage between under tree locations and off‐tree locations for sampling sites in the unfenced areas (Bray–Curtis Dissimilarity index = 0.84) than sites within the herbivore exclosures (Bray–Curtis Dissimilarity index = 0.41) (Figure 5c).

## 4 Discussion

Our study demonstrates that *A. drepanolobium* moderates the effect of herbivores on understorey vegetation community by creating refugia microhabitats. Generally, we found taller vegetation and more biomass underneath trees than away from trees canopies, and these differences were negatively correlated to tree canopy height. Areas beneath *A. drepanolobium* canopies were also associated with higher species richness. The difference in species composition between tree and off‐tree locations was greater in unfenced locations than within herbivore exclosures.

The observed differences in herbaceous vegetation height and biomass between tree locations and off‐tree locations suggest that areas underneath *A. drepanolobium* canopies experience reduced herbivory pressure. Similar “nurse plant” effects have been demonstrated elsewhere (Perea and Gil 2014; Manning et al. 2006; Scholes and Archer 1997; Belsky et al. 1989; Shannon and Morris 2001). The mechanism for such plant associations has often been linked to the nurse plants providing suitable microenvironments for germination and growth (Munguía‐Rosas and Sosa 2008; Schupp 1995), or protecting other plants from herbivory through chemical and olfactory deterrents (Feeny 1976; Finnerty et al. 2024; Tahvanainen and Root 1972), physical defenses such as thorns and spines (Coverdale et al. 2018), or caged architecture (García and Ramón Obeso 2003). Additionally, we suggest that symbiotic ants that protect trees against herbivores may mediate the nursing effect by *A. drepanolobium*. Being a highly palatable tree species (Birkett 2002), the survival of *Acacia drepanolobium* is strongly influenced by any symbionts (Goheen and Palmer 2010).

The relationship between nurse plants and the herbaceous vegetation growing underneath their canopies might even be mutually beneficial. First, increased herbaceous biomass can enhance soil quality through organic matter deposition (Navarro‐Cano et al. 2019) and improve nutrient cycling by altering soil microbiota (Lozano et al. 2014). Secondly, herbaceous vegetation cover, including tall grass, can protect the progeny of the nurse plant by obstructing the visual and physical access of herbivores (Riginos and Young 2007). Moreover, tall grass can create a microclimate that is less favorable for browsers, potentially deterring them from the area (Goheen and Palmer 2010). These protective effects of tall grass have been observed across various ecosystems, indicating it can potentially enhance tree seedling survival in the presence of herbivores. On the other hand, the increase in understory biomass may promote high‐intensity fire (Kimuyu et al. 2014) to the detriment of the nurse plants (LaMalfa et al. 2019; Ngugi et al. 2022; Werner et al. 2021). Our study demonstrated that trees with their canopies closer to the ground (saplings and coppicing trees) protect more herbaceous vegetation underneath their canopies than taller trees. However, shorter trees are also the most vulnerable to the frequent ground fires that characterize most arid and semi‐arid savannas (Hoffmann et al. 2019; LaMalfa et al. 2019). In addition to increasing fire risk, facilitated plants may intensify competition for nutrients and water. Riginos (2009) demonstrated that grass competition may limit the growth of *A. drepanolobium* as much as rainfall and fire.

The canopies of *A. drepanolobium* trees have a strong effect on biomass and composition of understory plant communities. In the presence of herbivores (outside the exclosures), under tree locations had remarkably higher herbaceous biomass and different species composition than off tree locations, but these differences were less pronounced in the absence of herbivores (inside the exclosures). These findings suggest that protection against herbivory by nurse plants can be crucial for the maintenance of a diverse community assemblage in areas facing chronic herbivory pressure. Similar herbivory‐mediated facilitation has been reported elsewhere (Cock and Hierro 2020; Graff et al. 2007; Rebollo et al. 2002; Verwijmeren et al. 2019). *A. drepanolobium* is one of the few tree species that thrives in heavy clay black cotton soils. To the extent that *A. drepanolobium* can protect other plant species from herbivory, areas underneath the trees may serve as refugia microhabitats. In chronically overgrazed systems, these refugia microhabitats may be important islands of biodiversity, providing a recolonization nucleus.

## Author Contributions

**Julius C. Karugu:** conceptualization (supporting), data curation (supporting), formal analysis (equal), investigation (lead), methodology (lead), writing – original draft (lead), writing – review and editing (supporting). **Duncan M. Kimuyu:** conceptualization (lead), data curation (lead), formal analysis (lead), methodology (supporting), project administration (lead), resources (lead), supervision (lead), writing – review and editing (lead). **David Kenfack:** resources (supporting), supervision (supporting), writing – review and editing (supporting). **Moshe Inbar:** conceptualization (lead), funding acquisition (lead), methodology (supporting), resources (supporting), supervision (supporting), writing – review and editing (supporting).

## Conflicts of Interest

The authors declare no conflicts of interest.

## Data Availability

The dataset supporting the conclusions of this article has been archived in dryad: https://doi.org/10.5061/dryad.k6djh9whb.
